# Stereotactic Radiotherapy for Localized External Auditory Canal Carcinomas: Report of Four Cases

**DOI:** 10.7759/cureus.14499

**Published:** 2021-04-15

**Authors:** Yoshimasa Mori, Shinichiro Mizumatsu

**Affiliations:** 1 Radiology and Radiation Oncology, Aichi Medical University, Nagakute, JPN; 2 Neurological Surgery, Ookuma Hospital, Nagoya, JPN; 3 Neurological Surgery, Aoyama General Hospital, Toyokawa, JPN; 4 Radiation Oncology and Neurological Surgery, Shin-Yurigaoka General Hospital, Kawasaki, JPN; 5 Cyberknife Center, Aoyama General Hospital, Toyokawa, JPN

**Keywords:** head and neck malignancies, hearing impairment, stereotactic radiotherapy, radiosurgery, squamous cell carcinoma, external auditory canal carcinoma, temporal bone, early stage

## Abstract

External auditory canal carcinoma (EACC) is sometimes diagnosed at an early stage because it arises superficially in the ear canal and may cause ear obstruction symptoms early. In addition, in the early stage of EACCs, involvement of lymph nodes or distant metastases are reported less frequently. And so, stereotactic radiotherapy (SRT) concentrating high-dose radiation on the primary tumor may be an effective option. The aim of this study is to evaluate the preliminary results of upfront SRT for early-stage localized EACCs. Four cases (four females, 84 to 98 years old) with EACC of N0M0 (=no lymph node involvement and no distant metastasis) were treated. All four tumors (0.30 - 11.1 ml in volume) were diagnosed as squamous cell carcinoma histologically. A total dose of 24 - 33 Gy in 3 - 5 fractions (D95 [dose delivered to 95% of the target volume]=100% dose) was delivered by SRT using CyberKnife. All four cases were alive at the end of the follow-up period of 19 to 106 months. In three cases (tumor volume, 0.3 - 3.5 ml) the treated tumors had regressed or disappeared by the end of the follow-up period of 106, 28, and 19 months respectively. In the remaining one case, the treated tumor (11.1 ml) regrew and cervical lymph node metastasis occurred, and both were treated with SRT again 6 months and 20 months after the initial SRT respectively. The tumors were still stable at 39 months after the initial SRT. In conclusion, in three cases the small tumors had regressed or disappeared without lymph node involvement or distant metastasis. In the remaining case, additional SRT was performed for recurrent tumors, after which the patient’s condition remained stable. SRT may be an effective option for early-stage EACCs.

## Introduction

External auditory canal carcinoma (EACC) is sometimes diagnosed at an early stage because it arises superficially in the ear canal and may cause ear obstruction symptoms early. In addition, in the early stage of EACCs, involvement of lymph nodes or distant metastases are reported less frequently [1,2]. Therefore, stereotactic radiotherapy (SRT) concentrating high-dose radiation on the primary tumor may be an effective option. The aim of this study is to evaluate the preliminary results of CyberKnife® (CK) (Accuray, Inc., Sunnyvale, USA) SRT for early-stage localized EACCs instead of surgical extirpation.

This study was approved by the Ethical Committee Board of Shin-Yurigaoka General Hospital (20190520-2) and Aoyama General Hospital (19-02). The need for patient consent was waived.

## Case presentation

Four cases (four females, 84 to 98 years old) with EACC of N0M0 (=no lymph node involvement and no distant metastasis) were treated (Table 1).

**Table 1:** Four cases of early-stage external auditory canal carcinomas treated by CyberKnife hypofractionated stereotactic radiotherapy FU=follow-up, mos=months, Gy=Gray, fx.=fraction, LN=lymph node involvement, T1=tumor diameter <2 cm, T2=tumor diameter >2 cm and <4 cm, CR=complete response, disappearance of the tumor, MR=minor response, tumor volume reduction by <25%, PG=tumor progression

Case	Age / Sex	Side	Tumor vol. (ml)	Prescription dose	Repeat SRT	FU (mos.)	Results
1	84/F	left	0.3 (T1)	24 Gy/ 3 fx.	(-)	106	CR
2	85/F	left	2.3 (T1)	33 Gy/ 3 fx.	(-)	28	MR
3	98/F	right	3.5 (T1)	30 Gy/ 5 fx.	(-)	19	CR
4	85/F	left	11.1 (T2)	33 Gy/ 3 fx.	primary (6 mos), LN (20 mos)	39	PG

All four tumors (0.30 - 11.1 ml) were diagnosed as squamous cell carcinoma (SCC) histologically. A total dose of 24 - 33 Gy in 3 - 5 fractions (D95 [dose delivered to 95% of the target volume]=100% dose) was delivered by CK-SRT. All four cases were alive at the end of the follow-up period of 19 to 106 months (Table 1). In three cases (tumor volume, 0.3 - 3.5 ml) the treated tumors had regressed or disappeared by the end of the respective follow-up period of 106, 28, and 19 months. In the remaining one case, the treated tumor (11.1 ml) regrew and cervical lymph node metastasis occurred, and both were treated with CK-SRT again 6 months and 20 months after the initial CK-SRT respectively. The tumors had been stable until 39 months after the initial CK-SRT. All four cases had hearing disturbance before CK-SRT. In two (Case 1 and Case 2) of four, after CK-SRT, the obstruction of the external ear canal was released and hearing improved by half one year after CK-SRT.

Illustrative cases

Case 1: 84-Year-Old Female

**Figure 1:**
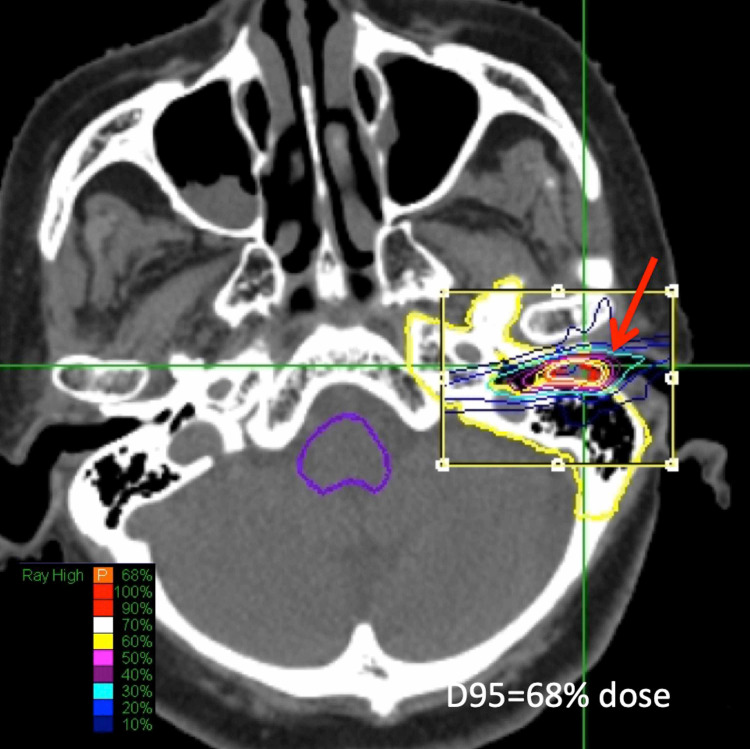
Dose Planning for Case 1 Axial image of iodine enhancement computed tomography (CT) on CyberKnife MultiPlan (Accuray, Tokyo, Japan) workstation showed excellent conformity for a small external auditory canal tumor (arrow). A prescription dose of D95%=24 Gy/ 3 fractions was adopted. After treatment, complete remission was obtained and maintained for 9 years.

Squamous cell carcinoma was identified by biopsy (Figure 1). A D95 (dose covering 95% of the tumor volume) prescription dose of 24 Gy in 3 fractions was delivered to a small tumor located at the external auditory canal by CK-SRT. After the treatment, complete remission was maintained until 9 years after the treatment.

Case 3: 98-Year-Old Female

**Figure 2:**
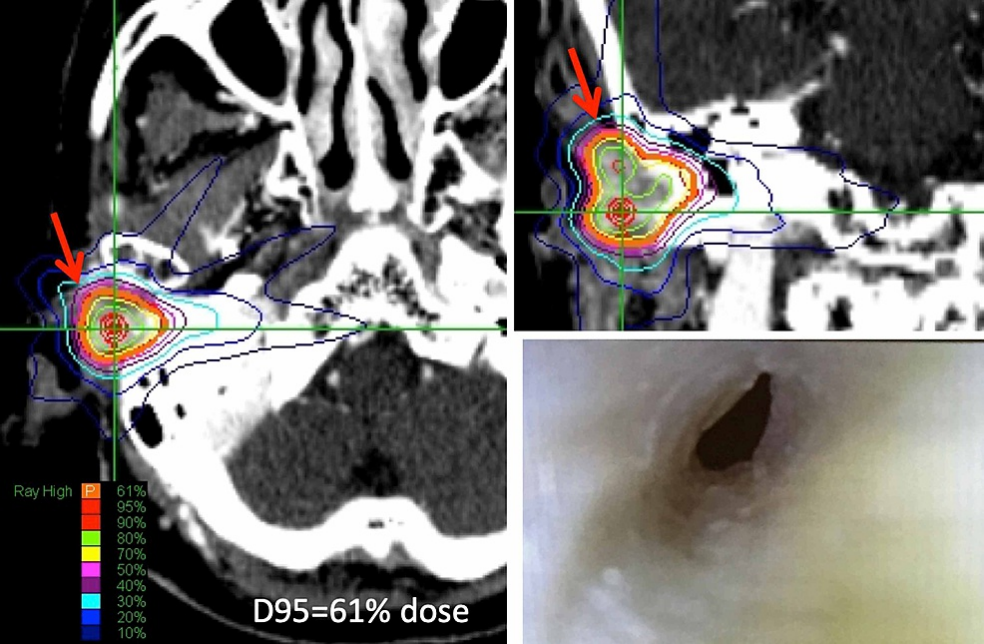
Dose Planning for Case 3 and Follow-up Axial (left) and coronal (right upper) images of iodine enhancement CT on MultiPlan workstation showed a small external auditory canal tumor (arrow). A prescription dose of D95%=30 Gy/ 5 fractions was adopted. Complete remission was obtained and maintained until 19 months after the treatment. Otoscopy (right lower) revealed patency of the external auditory canal 19 months after the treatment.

Squamous cell carcinoma was identified by biopsy (Figure 2). A D95 (dose covering 95% of the tumor volume) prescription dose of 30 Gy in 5 fractions was delivered to a tumor located at the right external auditory canal by CK-SRT. After the treatment, complete remission was maintained until 19 months after the treatment. Otoscopy revealed a patent external auditory canal at 19 months after the treatment.

## Discussion

Of various head and neck cancers, it is reported that the early-stage nasal and paranasal carcinomas [3-5] and early-stage external auditory canal carcinomas (EACCa) [1,2] rarely develop regional LN metastasis or cervical lymph node metastases [6]. Such patients might be good candidates for stereotactic radiosurgery (SRS)/SRT as an upfront therapy instead of surgical extirpation as the initial therapy [6].

Recently, Shinomiya et al. [1] reported the good results of surgical treatment of early-stage EACC in 33 cases (T1, 14; T2, 19). All were SCC and were operated on with sleeve resection or lateral temporal bone resection. In four of 33 patients, the surgical margin was positive and postoperative radiotherapy was added. The five-year overall and disease-specific survivals were 95% and 100% respectively. They described that potential parotid LN metastasis rates of T1 and T2 were 0% (0/14) and 5% (1/19) respectively. Regional recurrence in a parotid LN in a single case was successfully salvaged by total parotidectomy. They concluded that prophylactic superficial parotidectomy or neck dissection is not mandatory. Nam, et al. [7] also reported the results of surgery for EACCs. Locoregional recurrence occurred in four of 18 cases of T1 and T2.

Favorable results have been reported with SRT as the first-line therapy for auditory canal and middle ear cancers by Murai et al. [8]. These included T1 (n=3) and T2 (n=7). Doses of 37.5 Gy in 3 fractions or 40 Gy for 5 fractions were delivered as first-line therapy. The three-year overall survival rate and local control rate for T1/T2 disease were 69% and 70% respectively. Facial nerve function was preserved in all cases. In our present study, in all three cases of small tumors (T1) regression or disappearance without lymph node involvement or distant metastasis was achieved. In the other case (T2) additional SRT was performed for recurrent tumors, with the patient’s condition remaining stable. Adverse effects, defined as the deterioration of symptoms without tumor progression, were not observed. SRT may be an effective option for early-stage EACCs. Regarding the prescription dose, a more conservative regimen of 30 Gy in 5 fractions was adopted in Case 3, as we wanted to avoid toxicities considering her very old age (98 years old). In addition, a little reduced dose of 24 Gy in 3 fractions was adopted in Case 1 who was treated initially. In the other two cases (Case 2 and Case 4), we gave 33 Gy in 3 fractions, which was close to that reported by Murai et al [8]. The organs at risk were the brainstem, brain, facial nerve, ipsilateral cochlea (if the hearing was expected to preserve), and skin. We considered dose constraints (max point dose) provided by Timmerman [9], which were 20 to 24 Gy in 3 fractions. Except for the skin dose, they were easily achieved, as the tumors were not very large.

## Conclusions

In all three cases of small tumors (T1), regression or disappearance without lymph node involvement or distant metastasis was achieved. In the remaining case, additional SRT was performed for recurrent tumors and the patient’s condition remained stable. SRT may be an effective option for early-stage EACCs. A shorter treatment period of SRT would be beneficial, especially for elderly patients. Indications and treatment planning, including optimal prescription dose, fraction schedule, and field decision, will have to be established in future studies.
